# Childhood vaccination in rural southwestern Ethiopia: the nexus with demographic factors and women's autonomy

**DOI:** 10.11694/pamj.supp.2014.17.1.3135

**Published:** 2014-01-18

**Authors:** Yohannes Dibaba Wado, Mesganaw Fantahun Afework, Michelle J Hindin

**Affiliations:** 1 Department of Population & Family Health, College of Health Sciences, Jimma University, Ethiopia; 2 School of Public Health, Addis Ababa University, Ethiopia; 3 Johns Hopkins Bloomberg School of Public Health, Johns Hopkins University, USA

**Keywords:** Vaccination, immunization, women’s autonomy, pregnancy intention, antenatal care

## Abstract

**Introduction:**

Vaccination can reduce child mortality significantly and is a cost effective way to improve child health.Worldwide, more than 22 million children do not receive the basic recommended vaccinations.Vaccination coverage in Ethiopia remains low. Research on child health has focused on socio-economic factors such as maternal education and access to health care, but little attention has been given to demographic factors and women's autonomy within the household. The purpose of this study was to examine the influences of demographic factors and women's autonomy on the completion of childhood vaccination in rural Ethiopia.

**Methods:**

A cross-sectional community-based study was conducted in a Health and Demographic Survelliance System (HDSS) in southwestern Ethiopia. Data were drawn from a random sample of women with children aged 12-24 months (n = 889). Information on maternal socio-demographic characteristics and household variables were collected using an interviewer-administered structured questionnaire. Vaccination data were obtained from vaccination cards or mother's recall. Multivariate logistic regression was used to assess the association of independent variables with completion of childhood vaccination.

**Results:**

Of 889 children aged 12-24 months, 690 (78%) had received at least one vaccination. Only 37% (95% CI, 33.5-39.9) were fully vaccinated. Women's decision making autonomy, number of under-five children in the household, mother's education, use of antenatal care services and proximity to health facility were the main factors associated with full vaccination status.

**Conclusion:**

Completion of basic vaccination series is very low in the study area. Initiatives that enhance women's autonomy within the household and that promote healthy timing and spacing of pregnancies may help in improving child health through vaccination.

## Introduction

The fourth Millenium Development Goal (MDG 4) calls for efforts to reduce child mortality by two-thirds by 2015. Although there has been a decline in child mortality in recent decades in many regions of the world, about 7 million child deaths occur annually in the world [1]. The highest rates of child mortality occur in sub-Saharan Africa - where 1 in 9 children die before age five. The rate of decline in the region is also slower and most countries are unlikely to meet MDG 4 [1]. Ethiopia, one of the countries with high child mortality, has shown a remarkable decline in child mortality in the last decade. The under-five mortality rate declined from 166 deaths per 1000 live births in 2000 to 88 deaths per 1000 live births in 2011 [2, 3]. However, the majority of deaths are still caused by infectious diseases that can be prevented through cost-effective prevention efforts [4].

Childhood vaccination can reduce child mortality significantly and is a cost effective way to improve child health [5–7]. According to the World Health Organization (WHO), vaccination averts an estimated 2 to 3 million deaths every year from diphtheria, tetanus, pertussis, and measles [8]. In 2011, about 83 percent of infants worldwide were vaccinated with three doses of diphtheria-tetanus-pertussis (DTP3) vaccine, and 162 countries have reached DPT3 coverage of 80 percent. Globally, in 2011, 22.4 million children under one year of age did not receive DTP3 vaccination. Ethiopia is one of ten countries with large proportions of children without DPT3 vaccination [8].

The Ethiopian Health Policy emphasizes prevention and control of major communicable diseases [9]. Strengthening the Extended Program on Immunization (EPI) is therefore one of the core activities in the recent Health Sector Development Program (HSDP) to reduce child mortality. The EPI policy calls for BCG vaccine at birth, three doses of DPT-HepB-Hib vaccine at approximately 4, 8, and 12 weeks of age, four doses of oral Polio vaccine at 0-2, 4, 8, and 12 weeks of age, and measles vaccine at or soon after reaching 9 months of age. A child is said to be fully vaccinated if all eight vaccinations have been received. Although the EPI was initiated in 1980 with the goal of universal coverage by 1990 [10], achievements are still far below the international standard. According to the 2011 Ethiopian Demographic and Health Survey (EDHS), only 24% of children age 12-23 months were fully vaccinated. The coverage of measles vaccination was 56% [3] although service statistics by the Ministry of Health and a national EPI coverage survey conducted in 2006 showed a higher coverage than the DHS reports. The 2006 national EPI coverage survey showed that 50% of children age 12-23 months were fully vaccinated [11]. In addition to the low coverage, rural-urban and regional disparities in vaccination coverage are substantial [3]. The 2011 EDHS also showed that urban children were more than two times as likely as rural children to have all basic vaccinations.

Previous studies have documented several maternal, social and health care provision factors that influence completion of child vaccination in low and middle income countries. Maternal education and use of antenatal care services are consistently associated with completion of childhood vaccinations [12–16]. In studies from Ethiopia and other low and middle income countries, low access to services and inadequate awareness of the roles of vaccinations were found to be barriers to completion of child vacination series [10, 14, 17–19]. While the association between mother's age and parity and completion of childhood vaccination has been less consistent [14, 19, 20], two recent studies from Africa (Kenya and Nigeria) found that younger maternal age and lower parity are associated with completion of child vaccination series [15, 19]. Gender differences in vaccination coverage have been reported in studies from India [21], Bangladesh [22] and Nigeria [23] but not in previous studies from Ethiopia [14, 20].

Few studies in Sub-Saharan Africa have examined associations between vaccination rates and factors such as pregnancy intention, the number of under-five children in a family and women's autonomy within the household. However, these demographic factors are likely to be implicated in the coverage and completion of childhood vaccination. For instance, a large body of research highlights the negative consequences of unintended pregnancy on maternal health behavior. These studies have shown that unintended pregnancies are associated with delayed intiation and inadequate use of antenatal care services [24–26], maternal depression and anxiety during pregnancy [27, 28], and a shorter duration of breast feeding [29, 30]. On the other hand, studies that assessed the association between pregnancy intention and child preventive and curative care in high income countries found no effects [31, 32]. A study by Marston and Cleland (2003) that used DHS data from five low and middle income countries showed significantly higher risk of incomplete childhood vaccination for unintended births in three (Egypt, Kenya and Peru) of the five countries studied [33]. Another study that assessed the effects of unwantedness on curative care using DHS data from Indonesia showed that unwanted children were less likely to receive treatment for illness compared with wanted children [34]. Overall, there are few studies on the subject from low income countries and existing ones have shown mixed results.

Although some studies have examined the association between women's autonomy and maternal health care [35–37], few have examined whether a similar relationship exists in the utilization of child health services including vaccination. In the only study from Ethiopia that used the 2005 EDHS data to assess the associations of women's autonomy with maternal and child health care, women's participation in decision making was found to be significantly associated with completion of childhood vaccination [37]. Another study that assessed the associations of women's autonomy with under-five mortality from Central Ethiopia found that women's involvement in household decision making was significantly associated with under-five mortality [38]. This study examined the association between childhood vaccination and demographic factors including pregnancy intention, women's autonomy and the number of under-five children in a family in southwestern Ethiopia.

## Methods

### Study setting

A cross-sectional survey was conducted in the Gilgel Gibe Health and Demographic Surveillance System (HDSS) which is located 260 kilometers to the southwest of Addis Ababa (the capital) in southwestern Ethiopia. The Gilgel Gibe HDSS, which is run by Jimma University, is used to collect vital events data. The HDSS covers more than 10,000 households and a population of over 55,000 people.

### Sample

Women residing in the demographic surveillance area who had a live birth in the two years before the survey served as a sampling frame for the present study. The data used for this study were collected as part of a larger study on the effects of unintended pregnancy and related socio-demographic factors on maternal and child health in the HDSS. A sample size of 1,456 women was estimated for the study. Participants were drawn from eleven kebeles (smallest administrative unit in Ethiopia) in the HDSS area using simple random sampling. There were 1,370 women interviewed in the main study who gave birth to 1,382 children in the two years before the survey. A sub-sample of 889 children of age 12-24 months were eligible for the present analysis.

### Procedures

Data collection took place from March to May 2012. Data were collected by ten trained female data collectors who had a diploma-level training and data collection experience. They were closely supervised by supervisors who had similar or higher level of education and experience in supervision of data collection. The data collectors and supervisors participated in 5 days of training focusing on questionnaire administration and ethical considerations. After the training, a pre-test of the questionnaire was conducted. Information from the pre-test was used to finalize the questionnaire.

Data were collected using a structured questionnaire originally developed in English and translated to Oromo. Vaccination data were recorded from cards if the mother was able to present a card or reported verbally. All study participants were interviewed at their home in private area. Ethical approval was obtained from the College of Health Sciences, Addis Ababa University. Support letters were obtained from regional and district health offices. Local (kebele) administrations were informed about the study. Participants were briefed on the study and provided informed consent.

### Measures

The main outcome variable was full vaccination coverage of children age 12-24 months. We used the WHO definition of full vaccination which states that children are considered to be fully vaccinated when they have received a vaccination against tuberculosis (BCG), three doses each of DPT-HepB-Hib vaccine and polio vaccines, and a measles vaccination by the age of 12 months.

The main explanatory variables were women's pregnancy intention for the index child, number of under-five children in the household and women's participation in household decision making. Pregnancy intention was measured using the standard DHS approach, which asks women to recall their feelings at the time they became pregnant; “At the time you became pregnant, did you want to become pregnant then, did you want to wait until later, or did you not want to have any (more) children at all?” Women's participation in decision making was measured by asking the following questions; “who makes decisions in your household about: (1) obtaining health care for yourself; (2) large household purchases; (3) household purchases for daily needs; and (4) visits to family or relatives?” The responses were: (1) respondent alone, (2) respondent and husband/partner, (3) husband/partner alone, (4) someone else. Women were considered to participate in a decision if they usually make that decision alone or jointly with their husbands. A composite index was constructed by grouping women into two categories: women who participate in all four household decisions, indicating a higher level of autonomy, and women who do not have any say in one or more decisions. The internal consistency of the scale, as assessed using Cronbach's alpha, was 0.82. Socio-economic status was measured using a household assets index derived using principal components analysis. Maternal health seeking behaviour included antenatal care, place of delivery and postnatal check up. We also included several control variables including education, wealth index, parity, and distance from health facility.

Data analysis Data were analyzed using STATA software version 11. Bivariate associations between child vaccination and the explanatory and control variables were assessed using Chi-square analyses. At the multivariate level, two logistic regression models were run to identify factors associated with complete versus incomplete vaccination and receipt of at least one vaccination versus no vaccination. Variables were entered into the models based on their association in the bivariate analysis (at p < 0.20). Adjusted odds ratio and 95% confidence intervals are reported.

## Results

Sixty percent of mothers were aged 25-34 years, with a mean age of 27.5 years (SD±5.4) (Table 1).

**Table 1:** Description of study participants (N = 889), in Gilgel Gibe, southwestern Ethiopia, 2012

Socio-demographic Variables	Percent	Number of women
**Mother's age**		
15-24	24.6	219
25-34	59.5	529
35+	15.9	141
**Mother's marital status**		
Married	97.8	869
Divorced or widowed	2.3	20
**Religion**		
Muslim	92.5	822
Christian	7.5	67
**Mother's Educational status**		
No formal education	75.8	674
Primary	21.2	188
Secondary and above	3.0	27
**Residence**		
Rural	75.9	675
Urban	24.1	214
**Use of ANC during pregnancy**		
No ANC Visit	57.7	513
1-3 ANC visits	24.2	215
4 or more ANC visits	18.1	161
**Place of delivery for last birth**		
Home	88.6	788
Health facility	11.4	101
Pregnancy Intention		
Intended	65.0	574
Unintended	35.0	311
**Participation in decision making**		
Low	44.9	400
High	55.1	489
Mean number of living children	3.83	889

Almost all mothers were married (98%), most had no formal education (76%), were Muslim (93%), and lived in rural areas (76%). Women reported an average of four living children. Forty two percent of mothers made at least one antenatal care visit during their last pregnancy, but only 18% reported the recommended number of four or more antenatal care visits. Nearly nine in ten women delivered their last child at home. For 35% of the births, the pregnancy was reported as unintended. Fifty-five percent of women said they participated in all household decisions and were categorized as having high participation in household decisions.

**Figure 1:**
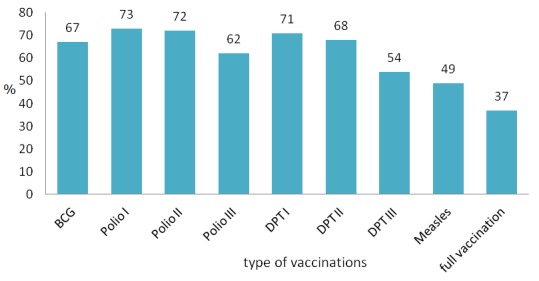
Percentage of children age 12-24 months who received different vaccinations series and full vaccination in Gilgel Gibe SW Ethiopia, 2012

Seventy-eight percent (n = 690) of children had ever been vaccinated. However, only 37% (95% CI, 33.5-39.9) of children age 12-24 months had received all basic recommended vaccinations (Figure 1).

Among the 690 children who had ever received vaccination, 86% received BCG, 92% received DPT1, 88% received DPT2, 70% received DPT3, and 63% received measles vaccinations (Table 2).

**Table 2:** Vaccination history of children age 12-24 months in Gilgel Gibe, southwestern Ethiopia, 2012

Variable	Frequency	Percentage
Any vaccination (n = 889)	689	77.6
BCG	596	86.4
DPT1	634	91.9
DPT2	604	87.5
DPT3	480	69.6
Measles	433	62.8
Vaccination card	283	40.9

Note: Percentages for the vaccinations and vaccination card variables are based on the number of those who had at least one vaccination

Almost half (48%) of those who had at least one vaccination received all basic vaccinations. Vaccination cards were produced for only 283 (41%) of children with at least one vaccination.

Table 3 shows bivariate associations between demographic and social factors and child vaccination (receiving at least one vaccination and full vaccination).

**Table 3:** Child vaccination by demographic and social factors, Gilgel Gibe, southwestern Ethiopia, 2012

Socio-demographic Variables	Received at least one vaccination	p-value	Fully vaccinated	p-value
**Mother's Age**		0.714		0.896
15-24	78.5		48.8	
25-34	78.7		46.7	
35 +	72.5		47.0	
**Sex of the child**		0.128		0.150
Female	76.0		49.4	
Male	79.3		45.1	
**Educational status**		0.010		0.012
No formal education	75.9		45.1	
Primary	81.4		52.9	
Secondary and above	96.3		57.7	
**Father's education**		0.003		0.01
No formal education	75.0		42.1	
Some education	84.0		55.8	
**Wealth tertile**		0.257		0.328
Low	74.8		46.5	
Middle	78.7		44.3	
Upper	79.4		51.1	
**Pregnancy Intention**		0.180		0.219
Intended	78.2		49.0	
Unintended	76.6		44.1	
**Participation in household decision making**		0.007		0.013
Low	72.7		43.9	
High	81.7	49.8		
**Number of under-five children**		0.008		0.023
1	79.5		52.5	
2	77.1		45.0	
3 +	70.4		31.6	
**ANC use**		0.001		0.008
None	68.8		42.3	
1-3 visits	87.0		48.7	
4 or more visits	93.7		57.4	
**Place of delivery**		0.001		0.033
Home	76.1		45.7	
Health facility	90.0		57.8	
**Walking distance from health facility**		0.001		0.001
≤ 60 minutes	84.9		53.7	
> 60 minutes	70.1		39.3	

The analysis showed that both receiving at least one vaccination and completion of vaccination varied by mother's education, women's participation in household decision making, number of under-five children in a family, antenatal care use, place of delivery and distance from health facility (p < 0.05). However, there was no variation in immunization (both completion and receipt of at least one) by mother's age, child's sex, wealth tertile and pregnancy intention.

Table 4 summarizes the results of the logistic regression analyses. Mother's age, wealth index and pregnancy intention were not significantly associated with vaccination status.

**Table 4:** Odds ratio from logistic regression predicting factors associated with child vaccination in Gilgel Gibe, southwestern Ethiopia, 2012

Variables	Receiving at least one vaccination^1^, OR (95% CI)	Completing all vaccinations^1^ OR (95% CI)
**Sex of the child**		
Female	Ref	Ref
Male	1.32 (0.94-1.88)	1.35 (1.00-1.82)*
**Mothers age**		
15-24	Ref	Ref
25-34	0.82 (0.54-1.24)	0.93 (0.61-1.44)
35 +	0.57 (0.28-1.14)	0.97 (0.52-1.78)
Mothers educational status No education	Ref	Ref
Primary	1.23 (0.86-1.69)	1.22 (0.87-1.55)
Secondary and above	2.74 (1.34-5.80)*	1.77 (1.04-3.59)*
**Wealth tertile**		
Poor	Ref	Ref
Middle	0.82 (0.54-1.24)	0.75 (0.51-1.11)
Rich	1.18 (0.71-1.95)	1.08 (0.72-1.63)
**Pregnancy intention**		
Intended	Ref	Ref
Unintended	0.81 (0.61-1.07)	0.93 (0.67-1.28)
**Number of under-five children**		
1	Ref	Ref
2	0.98 (0.64-1.51)	0.97 (0.68-1.39)
3 +	0.70 (0.33-1.46)	0.45 (0.21-0.93)*
**Father's education**		
Illiterate	Ref	Ref
Literate	1.20 (0.79-1.82)	1.38 (0.98-1.92)
**Participation in decisions**		
Low	Ref	Ref
High	1.63 (1.15-2.31)**	1.35 (1.01-1.80)*
**Distance from health facility**		
≤ 60 minutes	Ref	Ref
> 60 minutes	0.55 (0.38-0.80)**	0.58 (0.41-0.81)**
Antenatal care visits		
None	Ref	Ref
1-3 visits	2.79 (1.72-4.55)**	1.50 (1.06-2.13)*
4 or more visits	5.73 (2.77-11.84)**	2.27 (1.53-3.36)**
**Place of delivery**		
Home	Ref	Ref
Health institution	1.37 (0.63-2.98)	1.28 (0.80-2.03)

* Significant at P < 0.05

** Significant at P < 0.01

1 Adjusted for mother's age, education, wealth index, father's education, place of delivery, antenatal care use and distance from health facility

Male children were more likely than female children to be fully vaccinated. Among demographic factors, the number of under-five children in a family was significantly associated with completion of vaccination. Children are less likely to be fully vaccinated if there were three or more under-five children in the household (OR; 0.45, 95% CI, 0.21-0.96) compared with children living in households with only one under-five child. Women's participation in household decision making, maternal education, use of antenatal care services, and distance from the nearest health facility were associated with both completion of childhood vaccination and receiving at least one vaccination. Children were 1.35 times more likely to be fully vaccinated if their mothers participated in all household decisions than if they did not participate in all household decisions. Children with mothers who had completed secondary education were 1.77 times more likely to be fully immunized compared with children whose mothers had no formal education. Children were more likely to be fully vaccinated if their mother received any antenatal care during pregnancy. Children were 2.27 times more likely to be fully vaccinated if their mother reported four or more antenatal care visits than those whose mothers reported no antenatal visits while children whose mothers reported one to three visits were 1.5 times more likely to be fully vaccinated compared with those reporting no antenatal care visits.

Proximity to health facility, measured by the time taken to reach to the nearest health facility, was associated with full vaccination. Children from households living within a 60-minute walking distance from any health facility were more likely to complete vaccination schedules than those located farther than a 60-minute walking distance.

## Discussion

This study examined associations between childhood vaccination and demographic factors such as pregnancy intention, women's autonomy and the number of under-five children in a family in rural southwestern Ethiopia. We found that 78% of children age 12-24 months have received at least one of the vaccinination series, although only 37% completed all basic vaccinations. The proportion of children age 12-24 months who were vaccinated with DPT3 was 54%. Considering the fact that DPT3 is an indicator of the global Universal Childhood Immunization initiative, this level of DPT vaccination in the study site is quite low compared with the global average of 83% coverage [8]. The 2011 EDHS also showed that only 24% of children 12-23 months received all basic vaccinations and 37% received DPT3 vaccine [3]. These results show that the coverage and completion of basic vaccinations in Ethiopia is low, particularly in rural areas where the majority of the population lives.

We found that children from families with more than one under-five child were less likely to be fully vaccinated. This may be because women with many under-five children face a higher burden of care and may not be able to take their younger child(ren) for vaccination services. Other studies from low and middle countries have also found an association between parity and vaccination status [15, 19]. Pregnancy intention was not associated with vaccination status in this study showing that children from unintended pregnancies are no different from intended births in receiving full vaccination. Studies that assessed the association between pregnancy intention and child preventive and curative care in high income countries also found no effects [31, 32]. However, in a study by Marston and Cleland that used DHS data of five developing countries, unintended pregnancy was associated with incomplete childhood vaccination [33].

Child's sex was associated with fully vaccinated status with male children being more likely to be fully vaccinated than female children. Although previous studies on child vaccination did not report significant differences by gender in Ethiopia [14, 20], other studies have reported that significant gender differences exist in food consumption and schooling [39], and food insecurity and morbidity in Ethiopia [40]. There is a tradition of son preference in Ethiopia (44) and the current finding may thus reflect this tradition of sex preference in providing proper care for male children including the decision to immunize a child.

In Ethiopia, women are considered to be subordinate to men as evidenced by attitudes towards wife beating, women's participation in decision making, and women's financial autonomy [3]. We found that women's participation in household decision making was associated with complete vaccination. Participation in decision making on health care use (a dimension of women's autonomy) may enable women to independently or jointly decide to have their child vaccinated. Previous studies from Ethiopia [21] and Nigeria [23] also found that women's autonomy is important in the utilization of child vaccination services.

Our results show that other variables such as maternal education and use of antenatal care during pregnancy were significantly associated with full vaccination status. Children with mothers who have at least secondary level of education were more likely to complete the recommended vaccination series than children with mothers with no formal education. Education increases awareness on the role of vaccination services, and such awareness is important in influenceing use of vaccination services. This association is consistent with findings of several previous studies on maternal education and completion of vaccination series [12, 13, 15, 16, 20].

As observed in previous studies [13, 14, 20], use of antenatal care during pregnancy was significantly associated with completing childhood vaccination. Importantly, completing the recommended ANC visits (four or more visits) was strongly associated with full vaccination. The use of antenatal care encourages the use of subsequent maternal and child health services including vaccination [41, 42]. The use of delivery care was not significant in this study probably because the small number of women who delivered in health facilities meant the study was insufficiently powered for this analysis. Proximity to health facility, however, was associated with completion of the recommended vaccination series. This finding is consistent with previous studies from Ethiopia and other low and middle income countries, indicating that access to health facilities is an important factor for the utilization of child vaccination services [17, 43].

Study findings should be interpreted in light of several limitations. First, because this was a cross-sectional study, we cannot make causal inferences. In addition, although we sought to obtain vaccination data from actual records, not all women had vaccination cards for their children. As such, we had to rely on mothers’ reports which are subject to recall bias.

## Conclusion

Our study adds to the existing body of literature regarding the factors that influence childhood vaccination in low and middle income countries. Study findings highlight several potential avenues to improve childhood vaccination rates. First, the association between women's decision making autonomy and vaccination highlights the need for initiatives that improve women's autonomy in order to attain both gender equality and improved child health service utilization. In addition, in contexts characterized by low literacy levels, providing information and education about the benefits of childhood vaccination may be important. Antenatal care provides provide a good opportunity to provide mothers with information about vaccination and other maternal and child health services. Finally, improving access to family planning information and services is also important because healthy timing and spacing of pregnancies may ease the burden of care and hence promote health care use including vaccination.
